# Can hotels be used as alternative care sites in disasters and public health emergencies—A narrative review

**DOI:** 10.3934/publichealth.2024047

**Published:** 2024-07-25

**Authors:** Ruedeerat Khorram-Manesh, Amir Khorram-Manesh

**Affiliations:** 1 Hotel Management and Innovation Services, Nakhon Ratchasima Rajabhat University, Nakhon Ratchasima 30000, Thailand; 2 Institute of Clinical Sciences, Gothenburg University, 405 30 Gothenburg, Sweden; 3 Disaster Medicine Center (DMC), Gothenburg University, 405 30, Gothenburg, Sweden; 4 Gothenburg Emergency Medicine Research Group (GEMREG), Gothenburg University, 413 45, Gothenburg, Sweden

**Keywords:** alternative, care, disaster, public health, site

## Abstract

Managing disasters and public health emergencies poses a complex challenge, particularly in maintaining the crucial elements of surge capacity, often referred to as the 4S: staff, stuff, space, and system. While discussions surrounding the management of these emergencies typically emphasize their impact on emergency healthcare services, resources, and capabilities, it is essential to recognize the inherent limitations of these resources. Therefore, integrating non-medical resources such as community staff, supplies, and spaces into the response chain is equally important. Among community facilities, hotels are particularly intriguing due to their organizational and structural capabilities to serve as alternative care sites for lightly injured or non-injured emergency victims. This narrative review explored the potential use of hotels as alternative care sites and the legal implications associated with such utilization. The results confirmed a high potential for using hotels as alternate care sites. However, data concerning its practical and legal implications are insufficient. This paper suggests further research to investigate the criteria for utilizing hotels in this capacity, including admission guidelines for disaster victims and relevant ethical and legal considerations.

## 1. Introduction

Managing disasters and public health emergencies (DPHEs) presents a complex challenge, particularly in maintaining the essential components of surge capacity (SC), referred to as the 4S: staff, stuff, space, and system [1]. This aspect is pivotal in DPHE management because these events involve a dynamic process where resource needs can rapidly escalate or deescalate based on the severity and spread of the event [2]. The negative impacts of DPHEs span various sectors of society, affecting governmental centers, protective agencies, infrastructure, healthcare services, social service delivery mechanisms, and national sources of income such as tourism and the hospitality industry [3]–[5]. Addressing these consequences necessitates thorough planning and the implementation of contingency plans, emphasizing both individual entity planning and collaborative efforts to leverage all available societal resources through a holistic approach [6].

While discussions surrounding DPHE management predominantly focus on emergency healthcare service impacts, resources, and capabilities, it is crucial to recognize the finite nature of these resources. Once planned and reserved resources are depleted, there arises a need for additional staff, supplies, and space, initiating a second round of surge, which, if insufficient, results in a new search, prompting the development of new guidelines and systems to effectively manage the expansion of DPHEs by using other resources [2],[6],[7]. Therefore, despite the critical significance of medical aspects of DPHE management, non-medical considerations are equally important [8]–[10]. For instance, leveraging the community's non-medical resources to enhance flexibility in the capacity surge, *flexible surge capacity*, has been reported, advocated, and partly tested through simulation exercises and during real events [2],[6],[7].

Within the 4S framework, the need for staff and space might be more critical than the others in some situations. Whether within or outside hospitals, additional space is indispensable for treating and observing victims with various injuries or in need of psychological and social support [11],[12]. The concept of flexible surge capacity suggests, among others, the integration of dental and veterinary clinics into DPHE management strategies, a proposal that offers staff, stuff, and spaces and that has been evaluated positively by professionals [6],[7],[13]. Nevertheless, DPHE may damage available medical and paramedical spaces. Therefore, the possibility of using other spaces, such as sports arenas, hotels, and schools, for the care of victims as alternative care sites (ACS) has been highlighted [6],[13]. Among these ACSs, hotels have a special position due to their organizational structure and resources, i.e., staff, stuff (e.g., beds), and spaces [14]. In addition, most hotels have disaster plans to avoid physical and structural damage to their guests and the building. Shelters, evacuation, safety plans, educational initiatives to increase staff knowledge in DPHE management, and plans for business continuity are normally available in many hotels to safeguard against disaster impacts, especially in disaster-prone areas [15],[16]. Consequently, hotels might be a good space to care for uninjured or lightly injured disaster victims, alleviating pressure on medical facilities, as shown during the COVID-19 pandemic [2],[7],[17],[18].

Besides the willingness of emergency staff to participate in the chain of response, it is necessary to create educational initiatives to harmonize and facilitate seamless collaboration between different partners and agencies. Educational approaches in this context have been introduced and tested using collaborative factors, such as CSCATTT, which outlines immediate steps for achieving better outcomes in DPHE management [19],[20]. Derived from MIMMS courses (Major Incidents Medical Management and Support), CSCATTT encompasses factors such as command and control, safety, communication, assessment, triage, treatment, and transport [21]. Utilized in tabletop exercises, modular simulations, and real events, CSCATTT fosters collaborative partnerships among diverse agencies [19],[20]. For instance, it facilitates collaboration between agencies and communities, which have been encouraged to broaden their perspective and educate their members in basic disaster management, first aid, and basic life-support measures [22]–[24]. The individual skills gained from such initiatives increase society's resiliency and lead to higher preparedness in all parts of the community where its members contribute daily.

Although several published papers discuss the role of hotel resiliency during DPHE from a business continuity perspective, few delve into specifics about hotels' capabilities for the care of disaster victims, their effective integration into community response plans, and possible legal consequences of such integration [25],[26]. This study investigated whether a hotel can be used as an ACS and if any legal consequences may impact its engagement.

## 2. Materials and methods

### 2.1. Study design

This study aimed to gauge the breadth and depth of literature on the role of hotels in the DPHE response chain, thus aiming to provide a clear overview of the volume of available studies and their focus and offer valuable insights into emerging evidence. Starting as a systematic review according to Preferred Reporting Items for Systematic Reviews and Meta-Analyses (PRISMA) [27], a primary search was conducted to estimate the number of published articles based on research questions and search words. The result showed limited publications, preventing in-depth, quantitative, and qualitative analysis of the search and evaluation of the level of evidence. Consequently, the results of the systematic search were used to conduct a narrative review to summarize and interpret the current state of knowledge on the use of hotels as an ACS. Unlike systematic reviews or meta-analyses, which follow a specific and structured method for collecting and analyzing data, narrative reviews are more qualitative and descriptive [28]. This narrative review aimed to answer the following research questions: 1) Can a hotel be an alternative care site (ACS) and what can it be expected to do during DPHEs? and 2) What are the legal consequences of hotels participating in DPHE management?

#### 2.1.1. Search words

The search words *alternative*, *care*, *disaster*, *public health*, and *site* were used in isolation or combined.

#### 2.1.2. Search engines

The databases PubMed, Scopus, and Web of Science were used to search for available literature.

#### 2.1.3. Search strings

The search strings “Alternative” AND “Care” AND “Site” AND “Disaster” AND “Public health” were used in each database separately by entering each word to obtain the highest number of articles, combined with AND, and isolated by “search word.”

#### 2.1.4. Inclusion criteria

Studies encompassing the search words and discussing hotels as ACS with no time limitation and in English were included.

#### 2.1.5. Exclusion criteria

Studies that did not discuss hotels as ACS, reports from unreliable sources, and publications in other languages were excluded.

#### 2.1.6. Study eligibility

Studies were eligible for inclusion if they were reviews or original articles, and discussed alternative care sites, particularly hotels, during DPHE, and the legal aspects of ACS/hotel engagement in DPHE.

### 2.2. Review process

Both authors reviewed the title and abstract of each article independently. Dubious and undecided publications were sent to the second review round when both authors reviewed all selected articles together to achieve a consensus for the inclusion or exclusion of a paper. Not missing any important publications, the reference lists of included papers were also reviewed, and relevant studies were added for the final review.

### 2.3. Ethics approval of research

According to Swedish Law, where this study was performed, ethical approval is mandatory if the research includes sensitive data on the participants, e.g., political or religious views, or uses a method that aims to affect the person physically or psychologically (SFS 2003:460) [29]. This study did not cover these areas and was based on a review of existing and published articles.

## 3. Results

**Figure 1.**
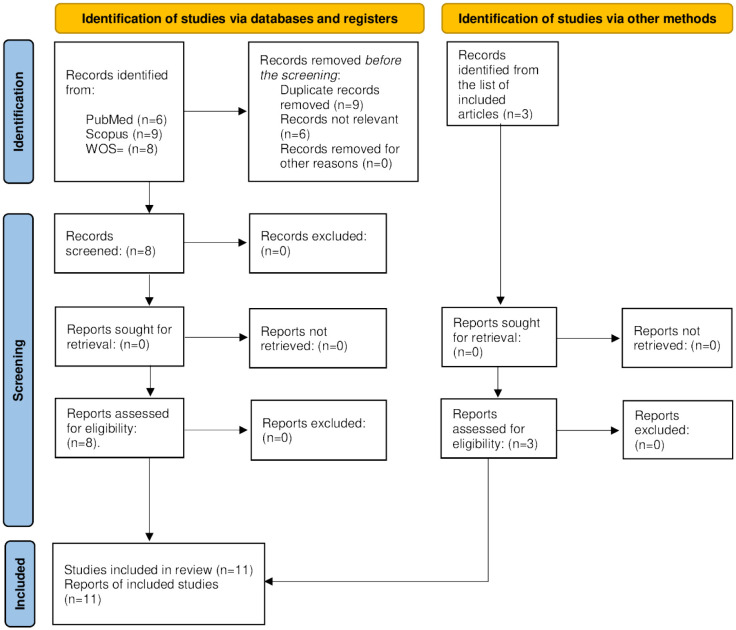
PRISMA 2020 flow diagram for new systematic reviews that included searches of databases, registers, and other sources [27].

The initial search yielded 23 publications across various databases: PubMed (6), Scopus (9), and Web of Science (8). Following the first review, 6 articles were found to be irrelevant based on predefined inclusion and exclusion criteria. Among the remaining articles, nine were duplicates, leaving 8 articles for a detailed second-round review. After a thorough evaluation, all eight papers in the second round of review were included. The review of reference lists of the included papers resulted in 3 relevant studies, which were also included (*n* = 11). Table 1 shows the summary of each paper, and Figure 1 the selection process. Due to the heterogenicity of the included papers, the evidence level could not be determined.

**Table 1.** Summary and characteristics of the papers included in this study [30]–[40].

**No**	**Author, Year, Journal**	**Summary**
**1**	Iserson, 2020, *Western J Emerg Med* [30]	Establishing ACS represents a critical step in disaster planning, transitioning communities from planning discussions to tangible actions that yield significant benefits during any disaster. This paper delves into essential considerations for selecting, establishing, and ultimately closing ACS. It addresses challenges such as administration, staffing, security, and ensuring the provision of essential supplies and equipment necessary for effective operation.
**2**	Rebmann, 2008, *J Perinat Neonatal Nurs* [31]	The article emphasizes recommended isolation practices for labor and delivery settings and provides guidelines for identifying and managing infected patients. It discusses potential outcomes for pregnant women and newborns affected by influenza and avian influenza. Pandemic planning considerations include ensuring hospital surge capacity, availability of medical equipment and staffing, and readiness to implement altered standards of care by communities as part of their disaster response plans to protect vulnerable populations.
**3**	Griffin et al., 2019, *Crit Care Nurs Clin* [32]	This article presents insights from a qualitative study on how the US Department of Veterans Affairs (VA) provided care for vulnerable veterans during Hurricane Sandy when medical centers were closed for an extended period. This experience underscores the ongoing need for care among vulnerable patients, even during and after disasters. Current hospital preparedness planning largely centers on sheltering-in-place or evacuation strategies. However, there is a need for research to understand how hospitals can deliver temporary emergency services in alternative settings, which can inform practical guidance for future preparedness efforts.
**4**	Yen et al., 2008, *J Formos Med Assoc* [33]	In addition to medical support, several important considerations should be incorporated into the successful design of alternative care sites (ACS). Namely: Utilize existing communication systems like the school's public address and information technology, along with mobile phones, for effective communication between nursing stations, patients, and families within the ACS. Ensure thorough checks and validation of facility security, including electrical safety, fire control, and environmental protection measures. Plan for potential shortages of medical personnel, which can reach high absenteeism rates (30%–40%). Develop a comprehensive training program for surge capacity, including utilizing retired medical personnel, medical and nursing students, and paramedics to bolster the pool of available personnel. Establish ACS as a critical component in building an infection control network, enhancing capabilities for the prevention and control of emerging communicable diseases.
**5**	Maslanka et al., 2022, *Disaster Med Public Health Prep* [34]	In March 2020, the Louisiana Department of Health activated the Medical Monitoring Station (MMS) in downtown New Orleans to provide relief to overwhelmed hospitals and nursing homes during the COVID-19 pandemic. Given the city's susceptibility to hurricanes, the MMS prioritized planning for potential tropical weather events. The planning process for hurricane preparedness at MMS involved collaboration across all sectors and agencies at the facility to ensure consistency and effectiveness. This resulted in the development of the MMS Shelter-in-Place Plan (MSIPP), which was complemented by a comprehensive tabletop exercise. The planning process highlighted six key topics essential for sheltering in place during hurricanes: patient care preparedness, interfacility coordination, wrap-around services, medical logistics, essential staffing, and incident command protocols. The MSIPP successfully enhanced patient safety and operational continuity during tropical storm Cristobal by activating specific components of the plan tailored to the threat level. This experience underscored the importance of originality, scalability, and flexibility in emergency operations planning during unprecedented events like the COVID-19 pandemic.
**6**	Walters et al., 2013, *Int J Disaster Resil Built Environ* [35]	To address the surge of patients during disasters, healthcare facilities need plans to quickly expand capacity. This paper proposes a concept for creating an independent, technologically advanced medical surge capacity using a convertible use rapidly expandable (CURE) center. To develop this concept, a scenario involving a large earthquake was explored at a selected site—an educational complex that was still under construction. By incorporating the required attributes into the design, the planning team envisioned a realistic solution. Challenges related to operations, communications, and technologies were identified and addressed to ensure the delivery of quality healthcare during disasters. Key findings from this process emphasized the need for community involvement, including experienced disaster response agencies or individuals. Analyzing regional threats and available resources is essential for effective planning, leading to a consensus on operational scope and site-specific needs. Computer modeling and virtual deployment of the center helped identify areas requiring additional planning.
**7**	Roszak et al., 2009, *Disaster Med Public Health Prep* [36]	Hospitals across the country implemented innovative approaches to manage the influx of patients due to the novel H1N1 virus. One effective strategy involves utilizing alternate sites of care, such as tents, parking lots, and community centers, to triage, stage, and screen patients, thereby alleviating pressure on emergency departments. However, even at these alternative sites, hospitals and healthcare providers must adhere to the requirements of the Emergency Medical Treatment and Labor Act (EMTALA). In this article, the authors examined the implications of EMTALA during public health emergencies, with a specific emphasis on its impact on alternative sites of care.
**8**	Bell et al., 2021, *Disaster Med Public Health Prep* [37]	During the COVID-19 outbreak, alternative care sites (ACS) in the United States were significantly underused despite the overwhelming burden of COVID-19 cases on the healthcare system, which also highlights the importance of surge capacity planning that considers multi-faceted demands. By reviewing current policies and literature on ACS, as well as drawing insights from the challenges posed by COVID-19, the authors offer recommendations to guide future surge capacity planning: 1) Continuously adapting and flexible preparedness actions, 2) addressing staffing needs promptly with pandemic-specific solutions, 3) prioritizing health equity in ACS establishment and planning, and 4) designing ACS to maintain safe and effective care standards.
**9**	Davis et al., 2021, *BMJ Simul Technol Enhanc Learn* [38]	Texas Children's Hospital (TCH) leveraged previous experience with alternative care sites (ACS) during surge events by redeploying mobile pediatric emergency response teams. To assess preparedness, they developed rapid simulation-based clinical systems testing (SbCST) with social distancing, involving collaboration between emergency management, pediatric emergency medicine, and the simulation team. They employed a two-phased approach: an initial virtual tabletop activity followed by in-person and virtual SbCST at each campus. Social distancing was strictly observed during these activities. Discussions used the Promoting Excellence and Reflective Learning in Simulation (PEARLS) method for debriefing, followed by compiling a failure mode and effects analysis (FMEA) disseminated to campus leaders. Over 2 weeks, participants from 20 departments identified 109 latent safety threats (LSTs) across four activities, prioritizing 71 as very high or high-priority items. Hospital leadership focused mitigation efforts on these priority threats. SbCST could rapidly refine pandemic responses and identify critical LSTs, allowing for virtual participation and social distancing within a condensed timeline. Prioritized FMEA reporting enabled leadership to efficiently address surge capacity concerns related to staff, supplies, infrastructure, and systems.
**10**	Matear, 2021, *J Bus Contin Emer Plan* [39]	During the COVID-19 pandemic in March and April 2020, a Federal Medical Station (FMS) was deployed in Santa Clara County, CA, to address healthcare needs beyond general shelters or acute care facilities. This study illustrates how the incident command system's flexibility allowed for combining the roles of situation unit leader and liaison officer at the FMS. This integration enhanced the FMS's effectiveness by improving situational awareness, information-sharing, and collaboration. While not universally applicable, merging such roles can be beneficial when skills and resources align, as demonstrated in this paper.
**11**	Scanlon et al., 2023, *Healthcare* [40]	During the COVID-19 pandemic, expanding healthcare capacity faced challenges beyond hospitals, necessitating a broader infrastructure network to handle rising infections and emerging cases. To ensure regional continuity of care, efforts focused on assessing buildings for alternative care sites (ACS) in non-healthcare settings. The American Institute of Architects (AIA) formed a COVID-19 ACS task force involving various professionals to create guidance during the pandemic's alert phase. They developed an ACS Preparedness Assessment Tool (PAT) to help healthcare teams quickly evaluate non-healthcare sites for healthcare operations. The tool was updated to V 2.0 and translated into multiple languages. The effectiveness of PAT V 2.0 was reviewed by the authors using case studies of healthcare teams establishing COVID-19 ACS in communities, recommending: 1) Policymakers should reconsider the built environment's role in the pandemic response for patients and healthcare workers; and 2) an updated ACS PAT tool should be a part of public health preparedness for healthcare surge capacity.

**Table 2.** Summary and characteristics of the papers included in this study [37],[38],[40].

**Requirements for ACS**	**Characteristics of a hotel matched to ACS**
**It must have a clear organization to make decisions (controls, opens, and closes ACS).**	Hotels have a classic up-to-down organization with a clear structure and direction of order. Necessary education in incident command systems may be provided yearly.
**It must have continuous and flexible preparedness actions (solutions for each scenario).**	Most hotels have a preparedness plan to follow during DPHE. Standardizing such plans to be used globally is needed and can be subject to future research.
**It must be able to promptly address staffing needs with specific scenario solutions.**	All hotels have a clear staffing plan in which diverse shifts are defined and reserves are planned. Extra resources can be called in and the shift can be longer if needed.
**It must be able to prioritize health equity in ACS establishment and planning.**	Most hotels can prioritize the needs of their guests according to the guests' physical condition and gender if needed. This suffices the need for equity in most cases.
**It must ensure ACS functionality without compromising care safety and effectiveness.**	There are safety and security functions at a hotel that can preferably be used to observe victims of a disaster. Adopting these functions for healthcare purposes needs research.
**It must be subject to continual reassessment and adjustments, to address management and treatment issues.**	Most hotels have small medical units. These units can be developed into larger entities with experts within the medical field and yearly control routines.
**It must have humanization plans to alleviate fear and enhance the patient's experience.**	Broadly, most 4 and 5-star hotels have these kinds of plans and programs. Future hotels might include these changes in their plans.
**It must have routine safety assessments to manage unforeseen changes and rapid response to operational challenges.**	Most hotels have private security units. However, they might need further education to understand other etiologies behind DPHEs.
**It must be able/plan to activate structural requirements, e.g., mechanical air and medical gas systems, electrical power, and potable water systems.**	Most hotels lack proper gas systems. However, systems concerning electrical power and potable water should be functional.
**It must be able to accommodate vulnerable populations and ensure cultural and language competency.**	Most people working with hospitality and hotel management are familiar with issues about vulnerable populations and cultural and language diversity.
**It must organize regular emergency drills to find and resolve issues related to space layout, equipment storage, communication, and staff training.**	Most hotels have yearly fire simulation exercises, which should be expanded to multi-hazards and multi-agency simulation exercises.
**There must be no legal issues, or if there are, they must be resolvable.**	There are broad legal directives regarding hotel management. Additional directives about the care of the victims can be issued locally.

### 3.1. Review results

After disasters such as the 1999 earthquake in Turkey and Hurricane Katrina in 2005, temporary medical facilities were rapidly set up in available spaces as alternate care sites (ACS), ranging from street corners to damaged buildings, with fewer concerns regarding purposefulness and safety [30]. In 2008, Rebmann emphasized the importance of earnestness and planning for ACS to provide safe and targeted care to vulnerable populations, including pregnant women and newborns [31]. Yet the same recommendation was again issued in 2019 by Griffin et al., who outlined the response of the US Department of Veterans Affairs to caring for vulnerable veterans post-Hurricane Sandy when medical centers were closed for an extended period. They emphasized the ongoing need for care among vulnerable patients during emergencies calling for further research on hospitals' delivery of temporary emergency services in alternative settings and developing practical strategies to address immediate and long-term patient needs [32].

The use of other spaces as ACS was highlighted by Yen & Shih in 2008, proposing the conversion of schools into designated ACS [33]. Following the 9/11 terrorist attack, Canadian emergency medicine residents utilized a New York City high school as a medical facility [30]. In New Orleans, the New Orleans Convention Center continued to function as a makeshift medical facility even months after Hurricane Katrina, serving thousands of patients monthly, many of whom lacked insurance coverage [34]. Nevertheless, the lack of valid criteria for ACS and adopting a simplified conceptual model like a stadium proved to be a real problem after DPHEs, necessitating detailed planning and discussions regarding ACS, including staffing, supplies, technologies, and protocols [30], and emphasizing that planning for ACS requires time, funding, and political support.

In a 2013 study, Walters et al. proposed the Convertible Use Rapidly Expandable (CURE) Center concept to provide independent, technologically advanced medical surge capacity [35]. They explored repurposing an educational complex as a potential CURE Center during a large earthquake. They also found operational, communication, and technological challenges, and stressed the importance of community involvement, regional threat analysis, and site-specific operational planning [35]. The same year, Roszak et al. [36] discussed the need for ACS in non-DPHE events, highlighting an increasing interest in setting up ACSs to alleviate emergency department (ED) overcrowding during flu seasons. They found that hospitals have two diverse options:

1) On-campus screening sites located anywhere designated by the hospital and staffed by qualified healthcare workers, who were eligible to conduct medical screening examinations based on symptoms to identify emergency medical conditions for further care or transfer to other departments without discrimination, ensuring immediate treatment for urgent cases, according to the Emergency Medical Treatment And Labor Act (EMTALA) obligations.

2) Off-campus sites, which were not dedicated emergency departments but staffed by the hospital, serving as screening sites in collaboration with the community. The authors emphasized further that community organizations could establish screening clinics without EMTALA obligations and recommended coordination with hospitals and emergency medical systems for further care and planning [36].

The emergence of the COVID-19 pandemic marked a new phase in discussions around ACSs, leading to many publications. Iserson [30] highlighted differences in creating diverse ACSs, noting China's rapid construction of new hospitals and other countries' deployment of military units and tent facilities, each with varying outcomes. In the US, states considered utilizing diverse facilities including hotels, ice rinks, stadiums, nursing homes, ships, and closed hospitals as ACSs. Iserson also raised some critical questions regarding the implementation of ACSs, particularly regarding control over site operation, usage criteria, facility selection, staffing challenges, security, supplies, equipment, and pharmaceutical access [30].

At the same time, Bell et al. [37] reported that during the COVID-19 outbreak, ACSs in the US were largely underutilized despite significant healthcare system challenges. They stressed the importance of surge capacity planning that considers diverse demands on response capabilities, drawing from current policies, past ACS literature, and COVID-19 experiences. Their recommendations included continuous and flexible preparedness actions, prompt addressing of staffing needs with pandemic-specific solutions, prioritization of health equity in ACS establishment and planning, and ensuring ACS functionality without compromising care safety and effectiveness. Similar recommendations about the functionality of ACS were given by Davis et al. [38], who reported on latent safety threats associated with establishing ACSs. They proposed simulation-based clinical system testing before the ACS establishment to identify, mitigate, or eliminate these latent safety issues [38]. In the same year, Matear [39] reported on creating a flexible incident command center (Federal Medical Station) close to the affected area in Santa Clara County, California, in 2020, to aid COVID-19 response efforts. Combining roles at the Federal Medical Station enhanced effectiveness and efficiency through improved situational awareness, information sharing, and collaboration. This report could add a new dimension to using other spaces during DPHEs.

Finally, Scanlon et al. described the development of the COVID-19 ACS Preparedness Assessment Tool (PAT) to assist local and regional stakeholders in evaluating non-healthcare settings for adaptive reuse as healthcare facilities during the pandemic in 2023 [40]. Released in April 2020 (V 1.0) and updated shortly after (V 2.0), the tool aimed to help all US states and territories prepare for suspected or confirmed COVID-19 cases. It provided architectural and engineering evaluation information synthesized from non-crisis situations, incorporating healthcare design criteria, best practices, evidence, and relevant codes and standards. Although not mandatory, V 2.0 guided a local cross-disciplinary team in strategically assessing ACS facilities, covering building selection criteria, operating parameters, program requirements, facility modifications, and considerations for vulnerable populations [40].

The authors stressed the need for ongoing assessment and adjustments at ACSs to address management and treatment issues, such as restroom accessibility and patient orientation due to limited natural light. They recommended implementing humanization plans to improve patient experience by offering outdoor spaces, family visitation rooms, wellness programs, and digital entertainment. Safety officers should make protocol adjustments to manage unforeseen challenges and ensure compliance during responses. Rapid response teams should handle deteriorating patient conditions and operational issues. Regular emergency drills were recommended to identify and resolve layout, equipment, communication, and training issues. Nevertheless, the main considerations remained to be admission criteria of victims, site selection, and alternative care sites' locations. Mechanical air, electric power systems, medical gas, and potable water systems were also necessary structural requirements for ACSs. Finally, addressing vulnerable populations and ensuring cultural competency, including the management of urban homelessness, and mental health needs, language skills, and knowledge about nutritional differences were reported as crucial elements in an ACS (Table 2) [40].

## 4. Discussion

While hotels possess the necessary organization and structure to be pivotal in all phases of disaster public health emergencies (DPHE), this review underscores that admission criteria and the medical condition of victims are the primary factors influencing their use during the DPHE response. These factors also dictate the type and scale of resources required and the legal implications [40]. An analysis of the necessity for an alternative care site (ACS), incorporating findings from current research and conclusions drawn from multiple studies [37],[38],[40], underscores the essential requirements for an optimal ACS. Table 2 illustrates how these requirements align with the potential characteristics of a hotel, encompassing organizational, structural, and environmental aspects. Many hotels possess structural advantages that make them suitable as ACS, offering rooms, beds, quarantine options, food and water resources, and areas for humanization. However, they may lack specific medical resources required for severely affected individuals, such as specialized air and gas systems. Consequently, hotels are well-suited for accommodating uninjured or lightly injured victims and those with social or psychological needs, thereby relieving strain on national, regional, or local healthcare systems during crises.

The concept of community engagement and flexible resource utilization in disaster management has been discussed for decades. The introduction of the flexible surge capacity concept in 2020 presented a new strategy to address all elements of surge capacity by collaborating with communities and utilizing their resources, including hotels, schools, sports arenas, dental clinics, and veterinary clinics as ACSs and part of the concept [6]. Later that year, Glantz et al. [13] tested this idea by surveying several hotels, schools, and sports arenas in Sweden to gauge their interest in taking part in DPHE responses and how they could assist their communities during such events. Five out of 10 hotels responded positively and expressed readiness to help during DPHE by accommodating uncomplicated, lightly injured, and mobile victims, and providing food, water, and shelter depending on their capacity. They also indicated a willingness to house evacuees and provide necessary items like blankets. Further education and training could enable them to undertake additional tasks, as confirmed by interviews with hotel managers [13].

In another study, Phattharapornjaroen et al. [24] built upon the survey conducted by Glantz et al. and investigated the willingness and capability of Thai hotels to assist disaster-affected individuals. Data was collected and compiled in 2020 during the initial waves of COVID-19, revealing interest among hotels in participating and providing assistance during DPHEs. Although the response rate was not as high as reported by Glantz et al., the returned responses indicated that hotels could serve as temporary housing facilities, offering water, food, shelter, childcare, and management of minor injuries. Notably, one hotel had employees trained in first aid and felt confident in providing more advanced assistance. However, all participants expressed a desire for further education.

The differences in response rates between the two investigations may be attributed to socio-cultural differences between Sweden and Thailand. Sweden is recognized for its collaborative culture, particularly in disaster management, where communities play a significant role and social and voluntary contributions are commonplace [41],[42]. As part of Swedish preparedness, there is legal backing for communities and emergency authorities to utilize buildings such as hotels as temporary care facilities. The “Förfogandeförordning” empowers emergency authorities to use various properties during crisis management and DPHEs [43].

Thailand is a tourist destination with a vast number of hotels that rely financially on their reputation and operational capacity. The projected growth of the hospitality industry in Thailand indicates a forecasted compound annual growth rate (CAGR) of 27.24% over the next five years (by 2029) [44], suggesting significant expansion driven by factors such as increased tourism, infrastructure development, and evolving consumer preferences. However, collaboration between diverse agencies, including non-governmental sectors, particularly in DPHEs, is not fully developed in Thailand. The lack of collaboration is one factor influencing the lower response rate observed in the study by Phattharapornjaroen et al. Additionally, Thailand had not yet been significantly impacted by COVID-19, and borders were closed during the study period, which may explain the lower response rate obtained in the Phattharapornjaroen study [24]. Nevertheless, the necessity of utilizing ACSs, particularly hotels, became more apparent in several countries during the COVID-19 pandemic. For instance, despite the absence of specific legislation [45], Thai healthcare authorities introduced the concept of “Hospitel,” converting hotels into hospitals. Collaborating with financially affected hotel and hospitality industry stakeholders, specific criteria were established for caring for COVID-19 patients in selected hotels [46]:

1. The hotel should have more than 30 rooms.
2. Eligible patients are those admitted and treated at the hospital, for at least 5–7 days, preferably under 50 years, neither children nor pregnant, with stable conditions, and normal/stable pulmonary X-rays.
3. Patients should be able to collaborate, communicate, and take care of themselves, should not be aggressive, and have no risks of developing psychiatric issues.
4. Patients should have no fever, and if they have any genetic condition, they should be able to handle their symptoms.
5. Patients must bring/have their medication, until attending physicians have a new treatment plan, if necessary.
6. The referring hospital should be willing and ready to readmit the patients with deteriorated medical conditions again for treatment and care if necessary.

Although large-scale disaster public health emergencies (DPHEs), such as mass casualty incidents and pandemics like COVID-19, often lead to the development of new legal guidelines for societal benefit [47], there currently exists no established legal framework supporting the provision of care to DPHE victims at hotels or other non-medical facilities during emergencies. During the COVID-19 pandemic, different countries adopted varied approaches, highlighting the lack of a standardized international response to the crisis. These national differences underscore the necessity for international rules and regulations, particularly in the standardized approach and management of DPHEs, given the increasing globalization and international tourism. Similar to existing regulations in health tourism [48], there is a need for comprehensive rules and regulations in disaster management.

Given the potential significant roles hotels can play in DPHE management, the absence of clear definitions and criteria for their use, and the lack of established legal implications and consequences for using hotels as ACS, there is a call for new discussions and the development of a standardized legal framework. This framework is essential to mitigate future complications associated with utilizing non-medical facilities, particularly hotels, in DPHE management.

## 5. Future research and recommendations

The requirements outlined in Table 2 represent a compilation of suggestions from various papers and should be subject to evaluation and validation in future studies. Experts could develop a practical scoring system, assigning points based on a hotel's possession, potential to develop, or lack of each characteristic. The threshold at which a hotel is deemed suitable to function as an ACS needs to be determined through new research. Previous experiences with patient hotels, established since the 1970s in several countries, may provide valuable insights. These purpose-built facilities accommodate patients with various medical conditions and are appropriately staffed and equipped [49].

Another crucial factor for using other facilities as ACS is the level of staff knowledge in first aid and basic life support. As previously described, one hotel in Bangkok had educated staff in these areas. Several studies have demonstrated that community members can be trained in medical and non-medical interventions to assist victims before emergency services arrive. These “immediate responders” are willing and capable of performing life-saving measures when necessary [22],[23]. Mandatory educational initiatives targeting staff in facilities like hotels should be implemented to enhance credibility and ensure guests' safety [5],[14].

Another point to be considered for future research is to evaluate the use of hotels in diverse types of disasters, which was not the aim of this study. There are several disaster classifications due to existing disagreement and changing characteristics of a disaster, focusing on hazards, indicating a paradigm change to hazards and risks assessment. This also resulted in the use of DPHEs in several publications since 2003 [50],[51]. DPHEs capture natural and man-made hazards and even other items in earlier classifications. It is also important to remember that public health emergencies can both be a consequence of a disaster and create a disaster themselves. Nevertheless, future research should focus on hazards and etiology and assess the feasibility of using hotels as ACS in each unique situation.

The legal and ethical implications of using specific facilities in the response chain during DPHEs require thorough examination. Legal considerations in using hotels as ACS are influenced by the extent of medical capabilities. Staffing hotels with appropriate medical professionals to meet victims' needs may suffice.

Finally, financial support should be extended to hotels that voluntarily participate in managing and caring for DPHE victims as part of the response chain. These upgraded hotels should be recognized for their efforts in ensuring guest safety and contributing as responsible members of society.

## 6. Limitations

The primary limitation of this study is related to its methodology. While narrative reviews are valuable for offering a comprehensive overview of a research area, integrating diverse perspectives, and providing insights for researchers and practitioners, they are susceptible to potential biases and limitations due to the subjective nature of synthesis and interpretation.

One challenge met was identifying suitable terms for a thorough search strategy, particularly when dealing with emerging or poorly defined literature, which could result in difficulties locating relevant papers. However, efforts were made to mitigate this risk by conducting a systematic search that included other sources such as reference lists of included papers, and undergoing multiple rigorous review rounds. These steps aimed to enhance the review's comprehensiveness, reliability, and transparency. However, although the number of obtained pieces of literature in a systematic review may not be crucial, the heterogenicity in compiled data on a new topic does not allow any evidence assessment according to a systematic review, resulting in a narrative review. SANRA has been suggested as a tool for assessing the outcome of a narrative review. However, in our opinion, it is very subjective to be used by authors, while it might work better for editors and reviewers [52].

## 7. Policy and practical recommendations

Although the use of hotels as ACS increases capacity, makes resource utilization more cost-effective, provides more private and comfortable accommodations compared to shelters and field hospitals, offers geographic flexibility, supports non-critical patients, and provides financial support to the struggling hospitality industry, several measures should be taken into consideration for policymakers and professionals. The following points offer some practical and policy recommendations:

1. Initiate a reference group of volunteer hotel management, policymakers, and medical and financial professionals to discuss the implications and implementation of hotels as ACS, based on each region's risks, possibilities, and limitations. Admission guidelines, the level of staff knowledge, hotel qualification guidelines, and financial incentives can be some areas for expert consideration.
2. Create routines for infection control in peacetime to increase preparedness and reduce the risk of disease spread in DPHEs. As part of this modification, other measures to adapt future hotels to meet healthcare regulations and standards should be discussed in a reference group (see above).
3. Equip selected hotels with basic medical equipment and supplies, and trained staff to manage basic life support and other necessary knowledge for basic care. This requires an increase in coordination, cooperation, and collaboration between volunteered hotels, healthcare, and other partners in the response chain to DPHEs.
4. Initiate clear communication with the public about using hotels as ACS and its pros and cons.
5. Continuous evaluation, assessment, and adjustment of policies and guidelines to improve the concept's effectiveness (see points 1 and 2). Training and educational initiatives for hotels' management, staff, and other response-chain partners enhance collaboration, ensure effectiveness, and can be used as assessment and evaluation tools.

## 8. Conclusions

The management of and response to disasters and public health emergencies require the mobilization of staff, resources (stuff), and physical space. To optimize response capabilities, new flexible strategies should be developed that leverage community resources through preplanned approaches guided by updated guidelines. Hotels are among the facilities that can be used to care for lightly injured or non-injured victims during emergencies. They have both structural and organizational attributes necessary for serving as alternative care sites. However, further studies are essential to investigate specific criteria for using hotels as alternative care sites. This includes defining admission criteria for disaster victims and addressing the ethical and legal requirements associated with utilizing hotels in this capacity. Such research is crucial for establishing standardized protocols and ensuring the effectiveness and appropriateness of utilizing hotels in disaster response scenarios.

## Use of AI tools declaration

The authors declare they have not used Artificial Intelligence (AI) tools in the creation of this article.

## References

[1] Bonnett CJ, Peery BN, Cantrill SV (2007). Surge capacity: A proposed conceptual framework. Amer J Emerg Med.

[2] Phattharapornjaroen P, Carlström E, Khorram-Manesh A (2022). Developing a conceptual framework for flexible surge capacity based on complexity and collaborative theoretical frameworks. Public Health.

[3] Khorram-Manesh A, Burkle FM, , (2020). Disasters and public health emergencies—Current perspectives in preparedness and response. Sustainability.

[4] Lorenzoni N, Stühlinger V, Stummer H (2020). Long-term impact of disasters on the public health system: A multi-case analysis. Int J Environ Res Public health.

[5] Henderson JC (2005). Responding to natural disasters: Managing a hotel in the aftermath of the Indian Ocean tsunami. Tour Hosp Res.

[6] Khorram-Manesh A (2020). Flexible surge capacity–public health, public education, and disaster management. Health Promot Perspect.

[7] Phattharapornjaroen P, Carlström E, Sivarak O (2022). Community-based response to the COVID-19 pandemic: Case study of a home isolation centre using flexible surge capacity. Public Health.

[8] Keim M, Abrahams J (2012). Health and disaster. Handbook of Hazards and Disaster Risk Reduction.

[9] Hugelius K, Becker J, Adolfsson A (2020). Five challenges when managing mass casualty or disaster situations: A review study. Int J Environ Res Public Health.

[10] Khorram-Manesh A, Lönroth H, Rotter P (2017). Non-medical aspects of civilian-military collaboration in the management of major incidents. Euro J Trauma Emerg Surg.

[11] Jayakody RRJC, Amarathunga D, Haigh R (2018). Integration of disaster management strategies with planning and designing public open spaces. Proc Engin.

[12] Mohr JA, Allen GM, Querry JL (2010). Healthcare facility disaster planning: Using GIS to identify alternate care sites. GIS in Hospital and Healthcare Emergency Management.

[13] Glantz V, Phattharapornjaroen P, Carlström E (2020). Regional flexible surge capacity—A flexible response system. Sustainability.

[14] Machado LP (2011). The consequences of natural disasters in touristic destinations: The case of Madeira Island – Portugal. Tour Hosp Res.

[15] Cheung C, Law R (2006). How can hotel guests be protected during the occurrence of a Tsunami?. Asia Pacific J Tour Res.

[16] Tsai CH, Linliu SC, Chang RC (2020). Disaster prevention management in the hotel industry: Hotel disaster prevention literacy. J Hosp Tour Manag.

[17] Dobie S, Schneider J, Kesgin M (2018). Hotels as critical hubs for destination disaster resilience: An analysis of hotel corporations' CSR activities supporting disaster relief and resilience. Infrastructures.

[18] Hidalgo A, Martín-Barroso D, Nuñez-Serrano JA (2022). Does hotel management matter to overcoming the COVID-19 crisis? The Spanish case. Tour Manag.

[19] Phattharapornjaroen P, Carlström E, Atiksawedparit P (2023). The impact of the three-level collaboration exercise on collaboration and leadership during scenario-based hospital evacuation exercises using flexible surge capacity concept: A mixed method cross-sectional study. BMC Health Serv Res.

[20] Sultan MAS, Carlström E, Sørensen JL (2023). Incorporating simulation exercises using collaborative tools into disaster and emergency medicine curriculum—A pilot survey among Saudi Arabian professionals. J Conting Crisis Manag.

[21] Sammut J, Cato D, Homer T (2001). Major incident medical management and support (MIMMS): A practical, multiple casualty, disaster-site training course for all Australian health care personnel. Emerg Med.

[22] Ashkenazi I, Hunt RC (2019). You're it—you've got to save someone: Immediate responders, not bystanders. Front Public Health.

[23] Khorram-Manesh A, Plegas P, Högstedt Å (2020). Immediate response to major incidents: defining an immediate responder!. Euro J Trauma Emerg Surg.

[24] Phattharapornjaroen P, Glantz V, Carlström E (2021). The feasibility of implementing the flexible surge capacity concept in Bangkok: Willing participants and educational gaps. Int J Environ Res Public Health.

[25] Brown NA, Rovins JE, Feldmann-Jensen S (2017). Exploring disaster resilience within the hotel sector: A systematic review of the literature. Int J Disaster Risk Reduct.

[26] Brown NA, Orchiston C, Rovins JE (2018). An integrative framework for investing disaster resilience within the hotel sector. J Hospital Tourism Manag.

[27] Munn Z, Peters MDJ, Stern C (2018). Systematic review or scoping review? Guidance for authors when choosing between a systematic or scoping review approach. BMC Med Res Methodol.

[28] Grant M, Booth A (2009). A typology of reviews: An analysis of 14 review types and associated methodologies. Health Inform Libraries J.

[29] Sveriges Riksdag (2003). Lag (2003:460) om etikprövning av forskning som avser människor.

[30] Iserson KV (2020). Alternative care sites: An option in disasters. Western J Emerg Med.

[31] Rebmann T (2008). Preparing for pandemic influenza. J Perinat Neonatal Nurs.

[32] Griffin AR, Gable AR, Der-Martirosian C (2019). Hospitals providing temporary emergency department services in alternative care settings after Hurricane Sandy. Crit Care Nurs Clin.

[33] Yen MY, Shih FY (2008). Transforming schools into pre-designed alternative care sites as part of preparedness plan for pandemic H5N1 influenza. J Formos Med Assoc.

[34] Maslanka M, Hurwitz JA (2022). An Eye on COVID: Hurricane preparedness at a COVID-19 alternative care site. Disaster Med Public Health Prep.

[35] Walters EL, Thomas TL, Corbett SW (2013). A convertible use rapidly expandable model for disaster response. Int J Disaster Resil Built Environ.

[36] Roszak AR, Jensen FR, Wild RE (2009). Implications of the Emergency Medical Treatment and Labor Act (EMTALA) during public health emergencies and on alternate sites of care. Disaster Med Public Health Prep.

[37] Bell SA, Krienke L, Quanstrom K (2021). Alternative care sites during the COVID-19 pandemic: Policy implications for pandemic surge planning. Disaster Med Public Health Prep.

[38] Davis NR, Doughty CB, Kerr T (2021). Rapidly building surge capacity within a pandemic response using simulation-based clinical systems testing. BMJ Simul Technol Enhanc Learn.

[39] Matear D (2021). Incident command system: Situation unit leader and county public health liaison roles during the federal medical station, Santa Clara, during the COVID-19 response. J Bus Contin Emer Plan.

[40] Scanlon M, Taylor E, Waltz K (2023). Evaluating efficacy of a COVID-19 alternative care site preparedness assessment tool for catastrophic healthcare surge capacity during pandemic response. Healthcare.

[41] Hjertzell C (2020). The magic Swedish ingredient for success is collaboration.

[42] Parker CF, Nohrstedt D, Baird J (2020). Collaborative crisis management: A plausibility probe of core assumptions. Policy Soc.

[43] Sveriges Riksdag (2023). Förfogandeförordning (1978:558).

[44] Mordor Intelligence (2023). Thailand hospitality industry size & share analysis. Growth trends & forecasts (2024–2029).

[45] PreventionWeb (2022). Thailand disaster management reference handbook 2022.

[46] Bangkok Biznews (2021). Take a look at the features of “Hospital” for COVID patients. How is it different from a field hospital?.

[47] Gostin LO, Friedman EA, Wetter SA (2020). Responding to COVID-19: How to navigate a public health emergency legally and ethically. Hastings Cent Rep.

[48] Aydin G, Karamehmet B (2017). Factors affecting health tourism and international health-care facility choice. Int J Pharm Healthcare Market.

[49] Quito A (2015). In Scandinavia, “patient hotels” provide an alternative to hospitals.

[50] Markenson D, DiMaggio C, Redlener I (2005). Preparing health professions students for terrorism, disaster, and public health emergencies: Core competencies. Acad Med.

[51] Lu S, Christie GA, Nguyen TT (2022). Applications of artificial intelligence and machine learning in disasters and public health emergencies. Disaster Med Public Health Prep.

[52] Baethge C, Goldbeck-Wood S, Mertens S (2019). SANRA-a scale for the quality assessment of narrative review articles. Res Integr Peer Rev.

